# How parental mediation affects adolescents’ problematic smartphone use: the chain mediating role of basic psychological needs and positive outcome expectations

**DOI:** 10.3389/fpsyg.2025.1590057

**Published:** 2025-07-21

**Authors:** Yan Chen, Qian Gu, Qishan Zheng, Biying Hu, Chuanhua Gu, Qingping Hu, Yuqi Cao

**Affiliations:** ^1^Tian Jiabing College of Education, China Three Gorges University, Yichang, China; ^2^School of Educational Science, Liaocheng University, Liaocheng, China; ^3^Key Laboratory of Adolescent Cyberpsychology and Behavior (CCNU), Ministry of Education, Wuhan, China; ^4^Key Laboratory of Human Development and Mental Health of Hubei Province, School of Psychology, Central China Normal University, Wuhan, China; ^5^Faculty of Education, University of Macau, Macau, China; ^6^Campus Hospital, Central China Normal University, Wuhan, China; ^7^School of Humanities, Political Science and Law, Henan University of Engineering, Zhengzhou, China

**Keywords:** parental mediation, problematic smartphone use, basic psychological needs, positive outcome expectations, Chinese adolescents

## Abstract

**Purpose:**

Problematic smartphone use can significantly jeopardize adolescents’ academic development as well as their physical and mental health. Although previous studies have explored the role of parental mediation strategies in adolescents’ problematic smartphone use, the underlying mechanisms remain unclear. This study aims to investigate the relationship between parental mediation and adolescents’ problematic smartphone use, with a specific focus on the chain mediation effects of basic psychological needs and positive outcome expectations.

**Methods:**

This study conducted a cross-sectional survey of 1,947 students from three junior high schools in Wuhan, Yichang, and Xiaogan, China, using a cluster sampling method. Validated scales were employed to measure parental mediation, problematic smartphone use, basic psychological needs, and positive outcome expectations.

**Results:**

The findings revealed that active mediation not only directly influences problematic smartphone use but also exerts an indirect effect through the individual mediation of basic psychological needs and positive outcome expectations, as well as through the sequential mediation from basic psychological needs to positive outcome expectations. Parental supervision not only directly influenced problematic smartphone use but also exerted indirect effects through the independent mediating role of basic psychological needs and the chain mediation pathway from basic psychological needs to positive outcome expectations.

**Conclusion:**

Active mediation and basic psychological needs significantly reduced adolescents’ problematic smartphone use, whereas parental supervision and positive outcome expectations significantly increased it. These findings provide theoretical insights into the mechanisms of problematic smartphone use and offer practical implications for educational strategies, emphasizing the importance of parents selecting appropriate mediation approaches, fulfilling basic psychological needs, and reducing positive outcome expectations to mitigate adolescents’ problematic smartphone use.

## 1 Introduction

Due to the rise of mobile digital media, adolescents now predominantly utilize smartphones for entertainment, gaining knowledge, and sharing ideas. According to the sixth national survey report on the use of the Internet by minors, smartphones are the primary internet access device for 97.3% of underage Chinese internet users.

Smartphone use has been found to positively impact adolescents’ academic growth, social interaction, leisure, and overall enjoyment (Abbasi et al., 2021). It’s important to be mindful, though, that prolonged and excessively uncontrollable smartphone use can potentially lead to problematic smartphone use (Fabio et al., 2022). Problematic smartphone use, also often referred to as smartphone addiction (e.g., Busch and Mccarthy, 2021), has garnered increased attention from researchers and public health practitioners in recent years (Busch and Mccarthy, 2021; Paterna et al., 2024; Ru et al., 2025; Sun and Tang, 2025; Tie et al., 2025). Problematic smartphone use is broadly defined as a compulsive pattern of smartphone usage which can result in negative consequences that impair the daily functioning of the user (Busch and Mccarthy, 2021). Compulsive use refers to an uncontrollable overuse characterized by maladaptive dependency and a tendency to use the smartphone without being separated from it. Specifically, for adolescents, negative consequences refer to the fact that problematic smartphone use adversely affects their sleep patterns, academic performance, and interpersonal relationships, and may lead to severe mental illnesses (Yang et al., 2020; Wacks and Weinstein, 2021; Mayerhofer et al., 2024).

Although the concepts of “smartphone addiction” and “problematic smartphone use “are often used interchangeably, some scholars (Panova and Carbonell, 2018) argue that the concept of “smartphone addiction” is controversial, as it has not been mentioned in either the DSM-5 or ICD-11 to date. Addiction is a disorder with severe effects on physical and psychological health. A behavior may have a similar presentation as addiction in terms of excessive use, impulse control problems, and negative consequences, but that does not mean that it should be considered an addiction (Panova and Carbonell, 2018). Panova and Carbonell (2018) suggest moving away from the addiction framework when studying smartphone usage behaviors and using alternative terms (such as “problematic smartphone use”) to describe them. Therefore, in this study, the terms “smartphone addiction” and “problematic smartphone use,” often used interchangeably, are consolidated and referred to as “problematic smartphone use.” Adolescents often struggle with problematic smartphone use more than adults do, largely due to their developing self-control (Fabio et al., 2022). Adolescents are especially prone to problematic smartphone use; nevertheless, adolescence presents a significant opportunity for personal growth. Hence, in addressing adolescents’ problematic smartphone use, it is of utmost importance to implement effective scientific prevention strategies and successful intervention methods.

### 1.1 The influence of parental mediation on problematic smartphone use

It’s important to recognize that adolescents often use their phones and other electronic devices at home, usually with their families. Parents have a vital role to play in guiding their children’s smartphone use, as they are their children’s first teachers and have a significant impact on their media education. Parental mediation involves the proactive steps taken by parents to mitigate the adverse effects of media on children and adolescents. These actions encompass a range of strategies that parents use to monitor, supervise, and provide guidance on the media and content to which minors are exposed (Fu et al., 2020; Martín-Cárdaba et al., 2024).

In the modern era of new media, researchers have outlined three key types of parental mediation:

(a) Active mediation, in which parents offer guidance on media content and usage through interactive forms such as discussions and explanations; (b) Restrictive mediation, where parents establish limits on their children’s media use; and (c) Parental supervision, involving parents keeping a close watch on their children’s media consumption, including checking emails and websites (Nikken and Jansz, 2014).

This study specifically focuses on two types of mediation strategies: active mediation and parental supervision. Whereas restrictive mediation is often the result of discussions and are set on a family level (e.g., there is a parental control software installed on the children’s device or not), active mediation and parental supervision can easily be applied individually and differently by each parent: each can choose (more freely than in the case of restrictions) how much they engage in active mediation and parental supervision (Dedkova and Smahel, 2019). As active mediation and parental supervision better represent parental mediation, this study mainly explores the impact mechanisms of these two aspects on problematic smartphone use.

Parental mediation theory underscores how parents strive to reduce the negative influences of media on their children (Clark, 2011). Through parental mediation strategies, children can take advantage of digital opportunities while being shielded from online dangers and risks (Durak and Kaygin, 2020). Social cognitive theory (Bandura, 1986) highlights the dynamic interplay among environmental factors, personal traits, and behaviors. As a key environmental factor, parental mediation plays a critical role in shaping adolescents’ smartphone behavior (Fu et al., 2020; Li and Liu, 2025). Active mediation encourages constructive dialogs between parents and teens about smartphone use, thereby strengthening the parent–child bond (Fu et al., 2020). Adolescents who enjoy close relationships with their parents tend to be more open in communication, sharing their thoughts, emotions, and behaviors more willingly. With parental active mediation, adolescents are guided toward a balanced perspective on smartphone use, helping them regulate overuse and adopt healthier digital habits (Fu et al., 2020). Thus, active mediation represents a practical and effective parental approach to addressing adolescents’ problematic smartphone behavior (Fu et al., 2020; Çiçek et al., 2021). Nevertheless, adolescents’ growing need for independence may cause parental oversight to backfire, triggering unintended negative effects (Li and Liu, 2025). For instance, excessive supervision might incite rebellious emotions, damage the parent–child relationship, convey distrust, and paradoxically increase the likelihood of problematic smartphone use (Gao et al., 2022).

As such, this study proposes the research hypothesis 1:

*H1a*: Active mediation is negatively associated with the problematic smartphone use.

*H1b*: Parental supervision is positively associated with the problematic smartphone use.

### 1.2 Mediating effect of basic psychological needs

According to self-determination theory (Deci and Ryan, 2000), human beings possess three basic psychological needs: the need for autonomy (the desire to regulate one’s own behavior without external coercion), the need for relatedness (the desire to establish and maintain close relationships with others), and the need for competence (the desire to feel effective in dealing with important tasks). The fulfillment of these basic psychological needs constitutes essential “nutrients” for healthy individual development. When the environment in which an individual resides fails to satisfy these needs, maladjustment may occur, or the individual may turn to alternative contexts to seek fulfillment. From this perspective, the satisfaction of basic psychological needs is not only an “outcome” influenced by the environmental context, but also serves as an internal “motive” that drives compensatory behaviors when such needs are unmet (Gao et al., 2022). According to self-determination theory, supportive parenting approaches help meet adolescents’ basic psychological needs, encouraging trust formation and the growth of positive behavioral patterns (Deci and Ryan, 2000). In contrast, unsupportive parenting may hinder adolescent development by failing to offer emotional warmth, adequate support, and empathy. This lack of support may drive adolescents to turn to the internet for psychological fulfillment as a way to cope with feelings of rejection in real life. Prior research indicates that emotionally warm and autonomy-supportive parenting nurtures adolescents’ sense of being accepted and empowered (Gao et al., 2022). These parenting practices contribute to the satisfaction of psychological needs, boost academic outcomes and well-being, and diminish externalizing tendencies (Deci and Ryan, 2000; Charlot Colomès et al., 2021; Abidin et al., 2022). Parenting characterized by psychological control and coercion is misaligned with adolescents’ core needs, as outlined in self-determination theory (Deci and Ryan, 2000). As a result, unmet psychological needs may drive adolescents to develop problematic patterns of smartphone use (Vossen et al., 2024).

Active mediation involves open, respectful conversations with children about both the benefits and drawbacks of smartphone use. These strategies reflect supportive parenting, encouraging empathy and emotional closeness (Liu et al., 2023). In contrast, strict supervisory approaches may be perceived as harsh, prompting adolescents to resist parental authority. This can strain parent–child dynamics and make constructive communication more difficult (Li and Liu, 2025). Such mediation styles resemble controlling parenting patterns and may obstruct the fulfillment of psychological needs. To date, little research has explored how distinct parental mediation styles affect adolescents’ psychological needs satisfaction. This raises the question of whether media-related parenting practices like mediation influence adolescents’ basic psychological needs fulfillment. Building on prior findings, this study hypothesizes that parental mediation strategies influence adolescents’ fulfillment of psychological needs. Specifically, active mediation is expected to enhance, while parental supervision may suppress, adolescents’ psychological need satisfaction. A key indicator of problematic smartphone behavior is the inadequate satisfaction of psychological needs in real-life contexts. Research shows that failure to meet basic psychological needs offline often leads individuals to seek fulfillment through excessive internet engagement (Chen et al., 2020). For example, social media may fulfill the need for relatedness, online gaming may satisfy the need for competence, while digital content may support the need for autonomy. In the digital era, smartphones, with their multifunctional apps, can readily satisfy users’ core psychological needs. This compensatory mechanism may drive smartphone overuse, potentially developing into problematic use patterns (Gao et al., 2022).

According to the self-determination theory, the satisfaction of basic psychological needs mediates the connection of the social environment and a person’s behavioral characteristics (Deci and Ryan, 2000). Numerous studies have shown that when individuals’ basic psychological needs are met, it not only links to positive outcomes like high well-being (Martela et al., 2022), self-esteem (Gong and Wang, 2023), proactive behavior, and better academic achievement (Liu and Huang, 2021) in supportive environments such as positive parenting and teacher support, but also serves as a buffer in unfavorable environments like high stress and controlling parenting, reducing negative outcomes like psychological distress, depression, anxiety (Peng et al., 2021; Liu et al., 2022), and behavioral problems (Ciydem et al., 2023). However, little research has been done specifically to investigate the mediating role of basic psychological needs in parental mediation and adolescents’ problematic smartphone use.

To summarize, this study proposes that innate basic psychological needs may act as a mediator in the relationship between parental mediation and adolescents’ problematic smartphone use. Therefore, hypothesis 2 was proposed in this study:

*H2a*: Active mediation is indirectly and negatively associated with problematic smartphone use of adolescents through basic psychological needs.

*H2b*: Parental supervision is indirectly and positively associated with problematic smartphone use of adolescents through basic psychological needs.

### 1.3 Mediating effect of positive outcome expectations

Positive outcome expectations (also known as network preference cognition) refer to individuals’ judgments regarding the potential positive outcomes of using the internet (Li et al., 2016). These judgments emerge in the initial stages of internet exposure and are reinforced throughout the process of internet usage, eventually becoming automated. As external stimuli typically need to be mediated through individuals’ cognitive interpretations, the perceived positive aspects of the internet may exert a more significant influence on behavior than the objective aspects of the internet environment (Li et al., 2016). Internet addiction revolves around maladaptive cognition, with positive outcome expectations playing a significant role in its development and maintenance, according to the cognitive-behavioral model of pathological internet use (Davis, 2001). Individuals with high positive outcome expectations seek additional online support, such as addressing their psychological needs and fostering positive interactions through mobile media. They often struggle to resist using social networking sites, leading to a higher likelihood of engaging in social media activities in general and an increased risk of developing addictions (Bhargava and Velasquez, 2021). Empirical researches have demonstrated that positive outcome expectations are a predictor of internet addiction (Jin and Jiang, 2025). However, there is limited empirical research on the relationship between positive outcome expectations and problematic smartphone use. Thus, this study hypothesizes that positive outcome expectations are positively associated with the problematic smartphone use.

Currently, no empirical research has explored the relationship between parental mediation and positive outcome expectations. According to the social cognitive theory (Bandura, 1986), human behaviors result from the interaction between the environment and the individual. Therefore, from the perspective of social cognitive theory (Bandura, 1986), parental mediation and positive outcome expectations, as important environmental and individual factors influencing adolescents’ smartphone use behavior (Fu et al., 2020; Jin and Jiang, 2025), may jointly affect adolescents’ smartphone usage behaviors. Empirical studies have also shown that individual characteristics, as well as environmental factors such as parental and peer influences, play an important role in shaping adolescent addictive behaviors (Gao et al., 2022). Although with age, adolescents’ time spent with peers increases and the role of parents in socialization gradually diminishes, in terms of media use, peer influence and parental influence differ. Peers do not have direct responsibility for the growth and development of their same-age counterparts (Nathanson, 2002). Unlike parental mediation, peers are less likely to engage in critical assessments of media content, and discussions among peers about violent TV programs may actually encourage violent behaviors in adolescents. Peer mediation is less likely to have a positive effect on individual media use (Nathanson, 2002). Gao et al. (2022) also found a significant negative correlation between the parent-adolescent relationship and adolescent problematic smartphone use, while the correlation between peer relationships and problematic smartphone use was not statistically significant. Therefore, parental mediation has a greater impact on adolescents’ media use than peer mediation. Previous studies have found that when adolescents engage in discussions and conversations with their parents about online games, it can alter their positive outcome expectations regarding online gaming (Kapetanovic et al., 2025). This form of parent–child interaction makes adolescents more sensitive to parental feedback and better internalize parental expectations (Liu et al., 2023), which also helps improve adolescents’ critical thinking (Liu et al., 2023), thereby reducing their positive outcome expectations for the internet. Therefore, active mediation may reduce positive outcome expectations in adolescents. Furthermore, individuals in adolescence have a strong sense of autonomy, and when parents adopt supervision strategies for adolescents’ smartphone use behaviors, adolescents are likely to experience conflicts with their parents (Li and Liu, 2025). Adolescents will evaluate conflict events and the real world, forming thoughts such as “Parents do not understand or care about me” and “The real world is really awful.” These thoughts, when coupled with immersion in the online world, are likely to form a strong contrast, leading to cognitive tendencies such as “The real world is inferior to the online world” or “I feel more respected and loved online,” which increases positive outcome expectations (Li and Liu, 2025). Therefore, parental supervision may lead to an increase in positive outcome expectations among adolescents.

Based on the aforementioned analyses, this study proposes that parental mediation may affect problematic smartphone use behaviors by acting as a mediator through positive outcome expectations. Hence, the present study proposes the following research hypothesis 3:

*H3a*: Active mediation is indirectly and negatively associated with problematic smartphone use of adolescents through positive outcome expectations.

*H3b*: Parental supervision is indirectly and positively associated with problematic smartphone use of adolescents through positive outcome expectations.

### 1.4 The chain mediating effect of basic psychological needs and positive outcome expectations

It is important to note that psychological needs that people cannot satisfy in real life may be compensated for by the content provided in cyberspace (Li et al., 2016). Yin and Shen (2023) proposed that individuals might form the belief that the online world is superior to the real world because they initially turn to the internet to fulfill psychological needs that are unmet in real life. Fulfilling our needs through the internet can become a repetitive cycle, potentially leading to internet addiction. Continuously fulfilling individual needs can lead to the development of internet addiction. Having positive expectations about the outcomes related to internet use, even though it’s not adaptive, might lead people to rely more on the internet to fulfill their psychological needs, potentially increasing the risk of internet addiction. Moreover, individuals tend to have higher positive outcome expectations when their basic psychological needs are not met in real life. This implies that unmet basic psychological needs could come before positive outcome expectations, and these two factors may interact to cause internet addiction (Li et al., 2016).

Social cognitive theory (Bandura, 1986) emphasizes the interaction between the environment, individual characteristics and behavior. Parental mediation, as an important environmental variable, has a significant impact on adolescents’ problematic smartphone use (Fu et al., 2020; Li and Liu, 2025). Moreover, basic psychological needs and positive outcome expectations may serve as important individual factors influencing adolescents’ smartphone usage behaviors (Chen et al., 2020; Jin and Jiang, 2025). According to the self-determination theory, the satisfaction of basic psychological needs mediates the connection of the social environment and a person’s behavioral characteristics (Deci and Ryan, 2000). Several studies have revealed that environmental factors can influence both basic psychological needs and positive outcome expectations, which are predictive of problematic smartphone use (Li et al., 2016). Moreover, in reality, the unmet basic psychological needs may precede the formation of positive outcome expectancies (Li et al., 2016). This study, leveraging social cognitive theory (Bandura, 1986) and self-determination theory (Deci and Ryan, 2000), aims to elucidate the impact of parental mediation on adolescents’ problematic smartphone use by examining the mechanisms of basic psychological needs and positive outcome expectations, from the perspective of the interplay between environmental and individual factors influencing behavior.

Based on the aforementioned analyses, this study proposes that basic psychological needs and positive outcome expectations may play a chain mediating role between parental mediation and problematic smartphone use. Thus, hypothesis 4 was proposed in this study:

*H4a*: Active mediation can be indirectly and negatively associated with problematic smartphone use of adolescents through basic psychological needs and positive outcome expectations.

*H4b*: Parental supervision can be indirectly and positively associated with problematic smartphone use of adolescents through basic psychological needs and positive outcome expectations.

**Figure 1:**
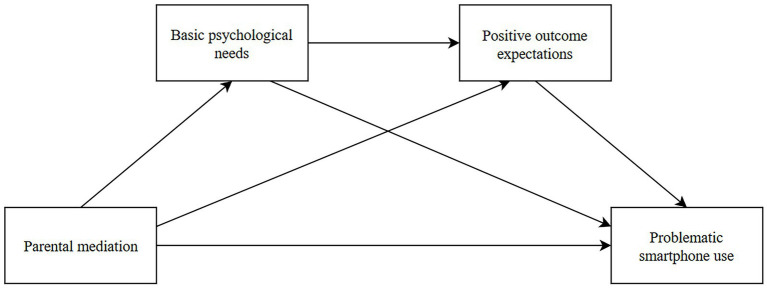
Diagram of the hypothesized model. Parental mediation includes active mediation and parental supervision.

Figure 1 graphically represents the model to be tested in the study.

## 2 Materials and methods

### 2.1 Participants

A total of three junior high schools from Wuhan, Yichang, and Xiaogan in China were selected using a whole cluster sampling approach. For each school, two classes of students from grades 7–8 were chosen, resulting in a sample size of 2,000 participants. After eliminating invalid samples (e.g., those with consistent responses across all questions, obviously fake answers, or missing answers to key variables), 1,947 valid questionnaires were recovered, yielding an effective recovery rate of 97.35%. The sample included 924 boys (47.46%) and 1,023 girls (52.54%), with ages ranging from 12.56 to 14.88 years (*M* = 13.72, *SD* = 1.16).

The study was conducted with the informed consent of school administrators, class teachers, and students. A web-based questionnaire survey was administered with the assistance of class teachers, using computer information technology during class sessions. A qualified individual holding a doctorate or master’s degree in psychology guided the participants throughout the process, providing clear instructions regarding the purpose of the questionnaire, the answering procedures, and emphasizing the voluntary nature of participation. The participants independently completed the survey within a time frame of approximately 20 min. Afterward, the completed questionnaires were collected and organized by the administrator. Ethical approval for the study was obtained from the Ethics Review Committee of Central China Normal University.

### 2.2 Measures

#### 2.2.1 Problematic smartphone use

In this study, the Smartphone Addiction Scale developed by Hong et al. (2012) was applied. This scale was adapted from the Young’s Internet Addiction Scale (Young, 1998) to gauge the extent of problematic smartphone use. Although the term “addiction” is used in the name of the Smartphone Addiction Scale, it is actually designed to measure the extent of “problematic smartphone use” as a continuous variable, without establishing a clinical diagnostic cutoff score. Therefore, it is appropriate to use this scale to assess problematic smartphone use. It included 11 items across three categories: time management and related issues, academic challenges and their repercussions, and reality substitution. For instance, “When using my smartphone, I always want to be able to play a little longer,” “My academic performance and concentration on my studies are affected by smartphone use,” “Before doing something, I always check my smartphone to see if there are any missed calls or unread messages” and so forth. The items are rated using a 6-point Likert-type scale, with anchors ranging from 1 (never) to 6 (always). The severity of the problematic smartphone use increases with a higher score. In this study, the Smartphone Addiction Scale had a Cronbach’s alpha coefficient of 0.89.

#### 2.2.2 Parental mediation

The updated Parental Mediation Questionnaire on Smartphone Use (Adolescent Version), comprises 20 items, dividing parental mediation into three categories: active mediation, restrictive mediation and parental supervision (Chen, 2021). As active mediation and parental supervision better represent parental mediation, this study mainly explores the impact mechanisms of these two aspects on problematic smartphone use (Dedkova and Smahel, 2019). The questionnaire uses a 5-point Likert-type scale, with 1 indicating “never” and 5 indicating “always.” Higher scores on each dimension indicate greater parental mediation. In this study, the questionnaire had a Cronbach’s alpha coefficient ranging from 0.81 to 0.90.

#### 2.2.3 Basic psychological needs

The Basic Psychological Needs Scale created by Deci and Ryan (2000) and updated by Yu et al. (2012) was also employed in this study. The scale consists of 21 items across three dimensions: autonomy need, competence need, and relatedness need. For instance, “I am usually able to express my ideas and opinions quite freely,” “Most of the time, I experience a sense of accomplishment from what I do,” as well as “I feel friendly with people I interact with on a regular basis.” All items are rated on a 5-point Likert-type scale, with 1 indicating “never” and 5 indicating “always.” Better total or mean scores indicate a greater degree of satisfaction of basic psychological needs. In this study, the scale had a Cronbach’s alpha coefficient of 0.91.

#### 2.2.4 Positive outcome expectations

The Perceived Preferences for Internet Use Scale created by Li et al. (2010) was used in this study. The 12-item scale gauges respondents’ perceived preferences for using the internet for social convenience, stress relief, and self-actualization. The scale uses a 5-point Likert-type scale, with 1 representing “not at all compatible” and 5 representing “completely compatible.” Higher scores represent a predisposition for favorable internet results. In this study, the scale had a Cronbach’s alpha coefficient of 0.92.

### 2.3 Statistical analyses

Initially, descriptive statistical analysis and Pearson correlation analysis were conducted using SPSS 26.0 to establish a preliminary understanding of the relationships among variables. To assess potential common method bias, a Harman’s single-factor test was performed. To examine the mediating effects of basic psychological needs and positive outcome expectations, 95% confidence intervals for indirect effects were estimated using the Bootstrap method with 5,000 resamples. Further analyses were conducted using the PROCESS macro (Model 6) in SPSS 26.0 to validate the serial mediation effect.

## 3 Results

### 3.1 Common method bias tests

Harman’s single-factor test was conducted in this study to measure common method bias (Podsakoff et al., 2003). The results revealed that there were 14 factors with eigenvalues greater than 1. The first factor accounted for only 14. 23% of the variance, below the critical threshold of 40%. Therefore, it can be concluded that there is no significant issue of common method bias in this study.

### 3.2 Descriptive statistics and correlation analysis of variables

This study conducted correlation analyses to examine the relationships among adolescents’ problematic smartphone use, basic psychological needs, positive outcome expectations, and parental mediation (active mediation, and parental supervision) after controlling for age and gender. Table 1 displays the means, standard deviations, and correlation matrices for each variable. The results showed significant positive correlations between active mediation and basic psychological needs (*r* = 0.38, *p* < 0.01), and significant negative correlations between active mediation and both positive outcome expectations and problematic smartphone use (*r* = −0.18, *p* < 0.01; *r* = −0.19, *p* < 0.01, respectively). They also revealed active mediation to be significantly and negatively correlated with parental supervision (*r* = −0.07, *p* < 0.01). Parental supervision showed a significant positive correlation with problematic smartphone use (*r* = 0.17, *p* < 0.01), and a significant negative correlation with basic psychological needs (*r* = −0.11, *p* < 0.01). As for the correlation between parental supervision and positive outcome expectations, it did not show statistical significance (*r* = 0.04, *p* > 0.05). Basic psychological needs exhibited a significant negative correlation with both positive outcome expectations and problematic smartphone use (*r* = −0.36, *p* < 0.01; *r* = −0.29, *p* < 0.01, respectively). Meanwhile, positive outcome expectations showed a significant positive correlation with problematic smartphone use (*r* = 0.38, *p* < 0.01).

**Table 1:** Descriptive statistics and correlation coefficient matrix for each variable.

Variables	*M ± SD*	1	2	3	4	5
1. AM	4.25 ± 0.87	1				
2. PS	2.69 ± 0.41	−0.07^**^	1			
3. BPN	3.54 ± 0.52	0.38^**^	−0.11^**^	1		
4. POE	2.53 ± 0.88	−0.18^**^	0.04	−0.36^**^	1	
5. PSU	2.98 ± 0.88	−0.19^**^	0.17^**^	−0.29^**^	0.38^**^	1

*N* = 1,947. M, mean; SD, standard deviation; AM, active mediation; PS, parental supervision; BPN, basic psychological needs; POE, positive outcome expectations; PSU, problematic smartphone use. Statistical significance is denoted as follows: ^*^p < 0.05, ^**^p < 0.01, ^***^p < 0.001 (the same below).

### 3.3 Chain mediating model test

The mediating effects of basic psychological needs and positive outcome expectations on the different types of parental mediation and adolescents’ problematic smartphone use were analyzed using the SPSS macro PROCESS.

**Figure 2:**
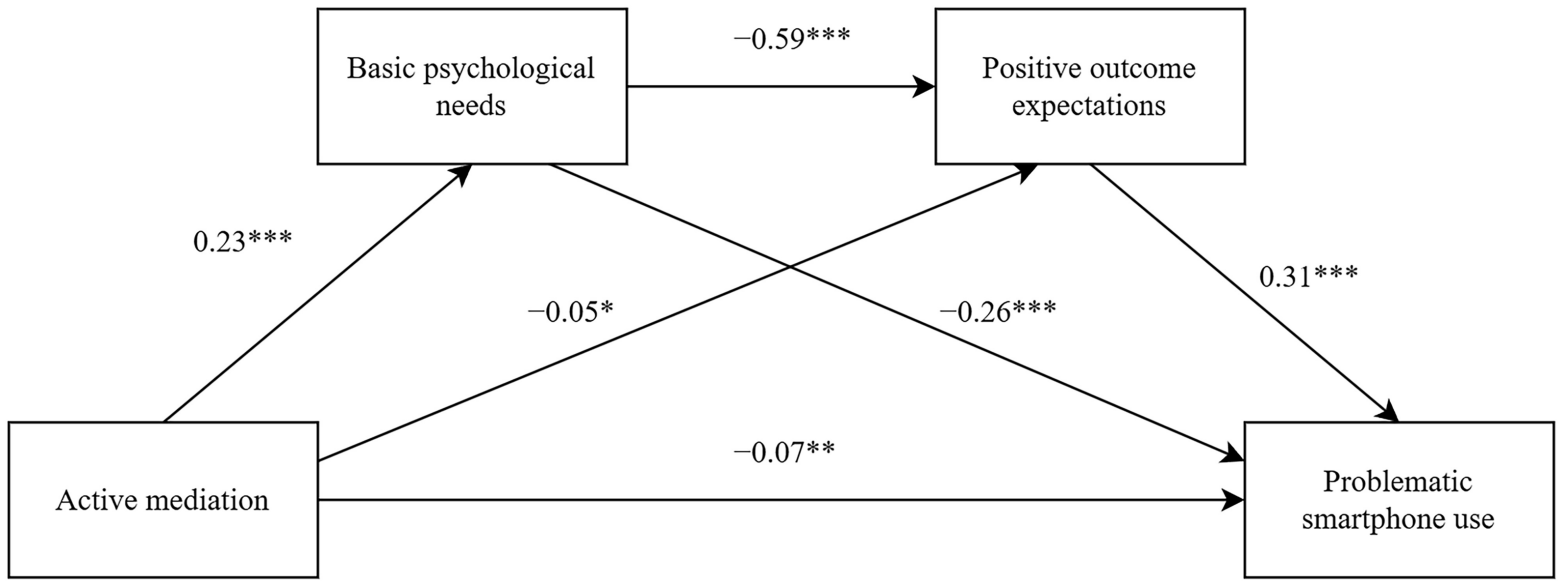
A chain mediating model diagram of active mediation and problematic smartphone use. * *p* < 0. 05, ** *p* < 0. 01, *** *p* < 0. 001.

Firstly, this study investigated how active mediation affects adolescents’ problematic smartphone use through the chained mediating role of basic psychological needs and positive outcome expectations. As shown in Table 2 and Figure 2, after controlling for age and gender, active mediation significantly negatively predicted problematic smartphone use and positive outcome expectations (*β* = −0.07, *p* < 0.01; *β* = −0.05, *p* < 0.05, respectively), and significantly positively predicted basic psychological needs (*β* = 0.23, *p* < 0.001). Basic psychological needs significantly negatively predicted problematic smartphone use (*β* = −0.26, *p* < 0.001) while positive outcome expectations significantly positively predicted problematic smartphone use (*β* = 0.31, *p* < 0.001). Basic psychological needs significantly negatively predicted positive outcome expectations (*β* = −0.59, *p* < 0.001). The study found the direct effect of active mediation on problematic smartphone use to be −0.07 (*t* = −3.20, *p* < 0.01, LLCI = −0.12, ULCI = −0.03), accounting for 36.84% of the total effect. The total indirect effect of basic psychological needs and positive outcome expectations on active mediation and problematic smartphone use was also noted to be −0.12, accounting for 63.16% of the total effect (detailed results in Table 3). Regarding the mediating effect of basic psychological needs, it was found to be −0.06, accounting for 31.58% of the total effect. Moreover, the bootstrap 95% confidence interval did not include 0, indicating a significant mediating effect of basic psychological needs on active mediation and problematic smartphone use. The indirect effect of positive outcome expectations on active mediation and problematic smartphone use was also found to be −0.02, accounting for 10.53% of the total effect. Moreover, the bootstrap 95% confidence interval did not include 0, indicating a significant mediating effect of positive outcome expectations on active mediation and problematic smartphone use. Meanwhile, the chained mediating effect of basic psychological needs and positive outcome expectations was determined to be −0.04, accounting for 21.05% of the total effect. The bootstrap 95% confidence interval did not include 0, indicating a significant chained mediating effect of basic psychological needs and positive outcome expectations on active mediation and problematic smartphone use.

**Table 2:** Regression analysis of the relationship between active mediation and problematic smartphone use.

Outcome	Predictor	*R^2^*	*F*	*β*	*t*
BPN	AM	0.15	333.72	0.23	18.27^***^
POE	AM	0.13	148.79	−0.05	−2.05^*^
	BPN			−0.59	−15.04^***^
PSU	AM	0.18	138.33	−0.07	−3.20^**^
	BPN			−0.26	−6.45^***^
	POE			0.31	14.11^***^

*N* = 1,947. AM, active mediation; BPN, basic psychological needs; POE, positive outcome expectations; PSU, problematic smartphone use.

**Table 3:** Mediating effects in the relationship between active mediation and problematic smartphone use.

Pathways	Effect	Boot SE	95% CI		Relative mediation effect
			Boot LLCI	Boot ULCI	
Mediating effect	−0.12	0.01	−0.14	−0.09	63.16%
AM → BPN → PSU	−0.06	0.01	−0.08	−0.04	31.58%
AM → POE → PSU	−0.02	0.01	−0.03	−0.01	10.53%
AM → BPN → POE → PSU	−0.04	0.01	−0.05	−0.03	21.05%

*N* = 1,947. AM, active mediation; BPN, basic psychological needs; POE, positive outcome expectations; PSU, problematic smartphone use.

**Figure 3:**
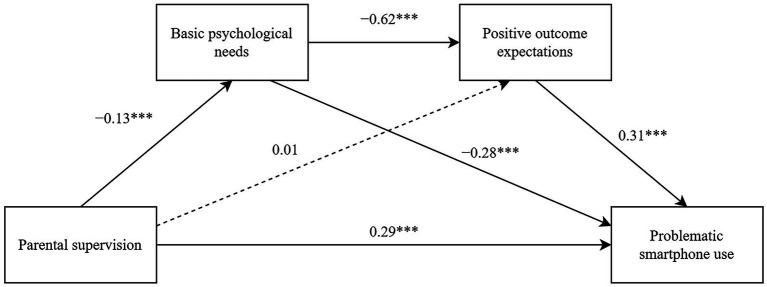
A chain mediating model diagram of parental supervision and problematic smartphone use *** *p* < 0. 001.

Finally, the impact of parental supervision on adolescents’ problematic smartphone use was investigated through the chained mediating effect of basic psychological needs and positive outcome expectations. As shown in Table 4 and Figure 3, after controlling for factors such as age and gender, parental supervision significantly positively predicted problematic smartphone use (*β* = 0.29, *p* < 0.001) and negatively predicted basic psychological needs (*β* = −0.13, *p* < 0.001), but it did not significantly predict positive outcome expectations. Basic psychological needs significantly negatively predicted problematic smartphone use (*β* = −0.28, *p* < 0.001) while positive outcome expectations significantly positively predicted problematic smartphone use (*β* = 0.31, *p* < 0.001). Basic psychological needs significantly negatively predicted positive outcome expectations (*β* = −0.62, *p* < 0.001). Meanwhile, parental supervision showed a direct effect on problematic smartphone use equal to 0.29 (*t* = 6.71, *p* < 0.001, LLCI = 0.21, ULCI = 0.38), which accounted for 82.86% of the total effect. Basic psychological needs and positive outcome expectations exhibited a total indirect effect on the impact of parental supervision on problematic smartphone use equal to 0.06, which accounted for 17.14% of the total effect (Table 5). The mediating effect of basic psychological needs was determined to be 0.04, accounting for 11.43% of the total effect. The bootstrap 95% confidence interval did not include 0, indicating a significant mediating effect of basic psychological needs on parental supervision and problematic smartphone use. Meanwhile, the indirect effect of basic psychological needs and positive outcome expectations on the impact of parental supervision on problematic smartphone use was found to be 0.02, accounting for 5.71% of the total effect. The bootstrap 95% confidence interval did not include 0, indicating a significant chained mediating effect of basic psychological needs and positive outcome expectations on the relationship between parental supervision and problematic smartphone use. However, positive outcome expectations did not mediate the relationship between parental supervision and problematic smartphone use.

**Table 4:** Regression analysis of the relationship between parental supervision and problematic smartphone use.

Outcome	Predictor	*R^2^*	*F*	*β*	*t*
BPN	PS	0.01	21.75	−0.13	−4.67^***^
POE	PS	0.13	146.39	0.01	0.11
	BPN			−0.62	−17.00^***^
PSU	PS	0.19	152.30	0.29	6.71^***^
	BPN			−0.28	−7.44^***^
	POE			0.31	14.38^***^

*N* = 1,947. PS, parental supervision; BPN, basic psychological needs; POE, positive outcome expectations; PSU, problematic smartphone use.

**Table 5:** Mediating effects in the relationship between parental supervision and problematic smartphone use.

Pathways	Effect	Boot SE	95% CI		Relative mediation effect
			Boot LLCI	Boot ULCI	
Mediating effect	0.06	0.02	0.03	0.10	17.14%
PS → BPN → PSU	0.04	0.01	0.02	0.06	11.43%
PS → POE → PSU	0.00	0.01	−0.03	0.03	
PS → BPN → POE → PSU	0.02	0.01	0.01	0.04	5.71%

*N* = 1,947. PS, parental supervision; BPN, basic psychological needs; POE, positive outcome expectations; PSU, problematic smartphone use.

## 4 Discussion

This study employed a chain mediation model to explore the relationships between parental mediation, basic psychological needs, positive outcome expectations, and adolescents’ problematic smartphone use. The findings revealed that active mediation can fulfill adolescents’ basic psychological needs, reduce positive outcome expectations, and ultimately lead to lower levels of problematic smartphone use. Additionally, the study demonstrated that both basic psychological needs and positive outcome expectations independently mediate the relationship between active mediation and problematic smartphone use. However, parental supervision was found to hinder the fulfillment of adolescents’ basic psychological needs, increase positive outcome expectations, and ultimately result in higher levels of problematic smartphone use. Furthermore, only basic psychological needs played a mediating role in the relationship between parental supervision and problematic smartphone use. The results of this study provide evidence for how parental mediation methods influence adolescents’ problematic smartphone use, while also offering empirical support for reducing problematic smartphone use and promoting mental health among adolescents.

### 4.1 The effect of parental mediation on adolescents’ problematic smartphone use

This study found that active mediation is negatively associated with adolescents’ problematic smartphone use, while parental supervision is positively associated with problematic smartphone use, confirming Hypothesis 1. The results indicated that providing proactive guidance to adolescents notably reduces problematic smartphone use, in accordance with prior empirical evidence (Fu et al., 2020). Active mediation refers to collaborative, open-ended discussions between parents and adolescents regarding media engagement. This dialog-centered approach has been found to foster adolescents’ critical thinking abilities (Liu et al., 2023), enabling them to more effectively navigate and resist the adverse effects of media exposure. Moreover, enhanced parent–child communication often reflects greater overall family functionality. Functionally cohesive families are more likely to prioritize their children’s educational development. Such parents are also inclined to offer enriched educational resources and cultivate a supportive home environment that satisfies adolescents’ core psychological needs. This can, in turn, decrease the risk of problematic smartphone use in adolescents.

Interestingly, the current findings suggest an unexpected positive relationship between parental supervision and adolescents’ problematic smartphone use, which stands in contrast to prior research. Previous studies have indicated that parental supervision can support adolescents in regulating smartphone use, improving time management, and promoting academic engagement, thereby potentially decreasing problematic use (Sun et al., 2022). However, according to psychological reactance theory (Brehm and Brehm, 2013), adolescents with a strong sense of autonomy tend to regard smartphone use as a personal issue. Excessive parental supervision may elicit feelings of psychological intrusion, prompting adolescents to resist, which could manifest as depressive symptoms, diminished self-efficacy, or maladaptive behaviors. Furthermore, during adolescence, individuals tend to become more private and less communicative with parents, driven by increasing desires for independence and self-regulation (Li and Liu, 2025). These developmental tendencies may cumulatively lead to escalated smartphone engagement, potentially culminating in problematic usage patterns. This study underscores the importance of adopting active mediation strategies as a more effective approach for guiding adolescents’ smartphone behaviors.

### 4.2 The mediating effect of basic psychological needs

The study found that basic psychological needs indirectly link parental mediation to problematic smartphone use of adolescents, confirming research on Hypothesis 2. To mitigate problematic smartphone use among adolescents, active mediation that caters to their basic psychological needs can be particularly effective. On the other hand, excessive parental supervision may impede the fulfillment of these needs, ultimately contributing to problematic smartphone use. The basic psychological needs theory (Deci and Ryan, 2000) states that effective parent–child interaction and communication can meet adolescents’ needs for autonomy and interpersonal satisfaction. When an individual’s fundamental psychological needs are effectively fulfilled, they will experience positive development and will be less likely to rely on the internet to satisfy these needs. This, in turn, can help prevent the development of problematic smartphone use. Furthermore, Kardefelt-Winther (2014) proposed a compensating model, suggesting that individuals may engage in excessive online behavior as a means of escaping real-life difficulties or regulating negative emotions. Parental supervision can undermine adolescents’ autonomy and fails to meet their needs for autonomy (Vossen et al., 2024). It also damages relationships between parents and children, neglects adolescents’ need for interpersonal interaction, and creates the perception in adolescents that their parents lack trust in their ability to handle technology, undermining their need for competence. Parental supervision strategies breed mistrust, disregard, and lack of understanding. This can lead adolescents to turn to the internet for guidance and support, which may increase their reliance on electronic devices, like smartphones, potentially leading to problematic smartphone use. Thus, it is evident that active mediation can reduce adolescents’ problematic smartphone use by fulfilling their basic psychological needs, whereas parental supervision may hinder the satisfaction of these needs, thereby increasing their likelihood of problematic smartphone use.

### 4.3 The mediating effect of positive outcome expectations

The study found that positive outcome expectations indirectly link active mediation to problematic smartphone use of adolescents, confirming research on H3a. However, this study did not find evidence to support the mediating effect of positive outcome expectations on the relationship between parental supervision and, therefore H3b was not confirmed. This study highlights that parental mediation can effectively mitigate adolescent problematic smartphone use by influencing their positive outcome expectations. In other words, actively involving parents in the use of smartphones can help reduce adolescents’ addiction by altering their beliefs about the positive outcomes of excessive smartphone use. However, positive outcome expectations did not mediate the interaction between parental supervision and adolescents’ problematic smartphone use. This could be because interactions between parents and children affect how adolescents view them. Active mediation, such as engaging in conversations with adolescents about their media use, can enhance their critical thinking skills (Liu et al., 2023). It can also reduce their expectations for positive internet outcomes, which in turn can help reduce problematic smartphone use among adolescents. When parents use supervision strategies, their children may feel like their personal freedom is being threatened. As a result, they might resist their parents’ advice and demonstrate unchanged positive expectations of the internet. When parents utilize supervision strategies, adolescents may not change their perceptions. Instead, these strategies may unintentionally prompt adolescents to engage in the restricted behaviors or to develop greater preferences for them. This phenomenon demonstrates the “forbidden fruit effect,” wherein the more taboo or offensive the content, the stronger the attraction to it. Adolescents might even trick their parents by looking for alternative ways to use the media (West et al., 2023). Therefore, this study found that active mediation can reduce adolescents’ problematic smartphone use by decreasing their positive outcome expectations. However, positive outcome expectations do not mediate the relationship between parental supervision and adolescents’ problematic smartphone use.

### 4.4 The chain mediating effect of basic psychological needs and positive outcome expectations

According to this study, basic psychological needs and positive outcome expectations of adolescents mediate the relationship between parental mediation and problematic smartphone use, confirming research on Hypothesis 4. Active mediation plays a crucial role in meeting adolescents’ basic psychological needs, thus helping to reduce positive outcome expectations and combat problematic smartphone use. Conversely, parental supervision hinders the satisfaction of basic psychological needs, thereby increasing positive outcome expectations and leading to problematic smartphone use. This result supports social cognitive theory (Bandura, 1986) and self-determination theory (Deci and Ryan, 2000). A possible explanation for this is that parents who engage in active mediation are adept communicators and are able to foster powerful connections with their children. A strong parent–child bond not only supports the basic psychological needs of adolescents, but also encourages them to accept their parents’ parenting style and level of education (Gao et al., 2022). This, in turn, lowers their expectations regarding internet use. When parents supervise their children’s smartphone use, they may feel that their autonomy and relationship needs are being violated, leading to rebellion. Unmet basic psychological needs can lead to individual psychological distress (Gu et al., 2024). This distress may result in heightened expectations for internet use and an increased risk of problematic smartphone use. Ladani et al. (2025) noted that individuals tend to develop addictions to specific social activities they engage in on their smartphones rather than the technology itself. For example, individuals may end up spending more time online than they expect while participating in interactive online activities such as gaming, chatting, and social networking, which increases the risk of developing addiction.

Overall, this study reveals the underlying psychological mechanisms through which parental mediation styles influence adolescents’ problematic smartphone use: parental mediation affects adolescents’ problematic smartphone use through the chain-mediating roles of basic psychological needs and positive outcome expectations. These findings confirm the mechanisms by which parental mediation impacts individual psychology and enrich research in the field of cyberpsychology. On one hand, it suggests that parents should adopt active mediation strategies to guide adolescents’ smartphone use. On the other hand, it emphasizes the importance of parents paying attention to adolescents’ smartphone use to minimize the potential harm of problematic smartphone use on their academic performance and mental health.

## 5 Implications, limitations, and future research

In this study, both motivational and non-adaptive cognitive aspects were theoretically integrated to examine the mediating role of basic psychological needs and positive outcome expectations in parental mediation, contributing to a clearer understanding of the underlying mechanism. It also offered a deeper insight into the interactions among different types of parental mediation, basic psychological needs, and positive outcome expectations. Accordingly, this study deepens our understanding of the pathways through which parental mediation influences adolescents’ problematic smartphone use. To support adolescents in using smartphones responsibly, it’s important for parents to engage in positive interaction with them, communicate openly, and explore the digital world together. This approach helps ensure that adolescents have their basic needs met and develop a balanced perspective on smartphone use, reducing the likelihood of seeking validation or success solely through online channels. In doing so, individuals can avoid developing an addiction to their smartphones. Parental supervision may hinder meeting psychological needs and forming positive outcome expectations from smartphone use. For example, when teenagers receive “likes” on social media platforms, it is considered a positive form of feedback (Da Silva Pinho et al., 2024). Believing that social media use will bring them joy may lead to problematic smartphone use. Moreover, parents can effectively help their children overcome negative thinking patterns by using active mediation techniques, rather than solely focusing on their anxiety levels. In summary, this study has constructed a theoretical model that delineates how individual factors, such as basic psychological needs and positive outcome expectations, are influenced by parental mediation within the family environment, thus affecting adolescents’ problematic smartphone use. This study contributes to the advancement of theories about parental mediation and offers crucial evidence to help prevent and address problematic smartphone use among adolescents.

However, the study has some limitations. This study suggests that while parents use parental supervision in their everyday lives, these actions may in fact increase the likelihood of adolescents developing problematic smartphone use. Some argue that parental mediation is a dynamic process arising from everyday interactions between parents and children, rather than a system of preset rules and techniques (West et al., 2023). Encouraging open communication and strengthening bonds with their adolescents are constructive ways for parents to regulate their internet use, given that smartphones are private and difficult for parents to monitor (Da Silva Pinho et al., 2024). The focus of future research should be on developing curricula that promote media literacy in adolescents. This should involve educating parents on using active mediation to enhance communication with their children, as well as guiding them on how to replace supervisory methods with active mediation (West et al., 2023). In addition, this study took a comprehensive approach to examining parental mediation by analyzing the combined methods used by both parents, instead of focusing solely on the individual roles of fathers and mothers. This allowed for a more thorough assessment of the effect of parental mediation on adolescents’ problematic smartphone use. Recent studies have been taking a closer look at the distinct effects that fathers’ and mothers’ mediation styles have on the media behaviors of adolescents (Boniel-Nissim et al., 2020), in contrast to earlier studies which examined parents’ mediation strategies as a whole (Beyens and Valkenburg, 2019). Consequently, there may be some differences in the effect of the mediation approaches employed by both parents on adolescents’ problematic smartphone use, and future studies should examine the relative contributions of father and mother mediation or the interaction of father’s mediation with mother’s mediation. Future research can also extend to examine the mechanisms by which parental mediation, positive outcome expectations, and adolescents’ basic psychological needs affect medical health risks such as abnormal changes in dopamine and cortisol levels associated with mobile phone addiction.

## 6 Conclusion

In conclusion, it was discovered that parental mediation is a crucial factor for adolescents’ problematic smartphone use. Additionally, current findings suggested that the relationship between parental mediation and adolescents’ problematic smartphone use may be mediated by basic psychological needs and positive outcome expectations. These insights provide a deeper understanding of how and why parental mediation can influence adolescents’ problematic smartphone use, offering empirical evidence and valuable intervention recommendations for future research on this topic.

## Data Availability

The raw data supporting the conclusions of this article will be made available by the authors, without undue reservation.

## References

[ref1] Abbasi G. A. Jagaveeran M. Goh Y. Tariq B. (2021). The impact of type of content use on smartphone addiction and academic performance: physical activity as moderator. Technol. Soc. 64:101521. doi: 10.1016/j.techsoc.2020.101521

[ref2] Abidin F. A. Yudiana W. Fadilah S. H. (2022). Parenting style and emotional well-being among adolescents: the role of basic psychological needs satisfaction and frustration. Front. Psychol. 13:901646. doi: 10.3389/fpsyg.2022.901646, PMID: 35783695 PMC9242003

[ref3] Bandura A. (1986). Social Foundations of Thought and Action: A Social Cognitive Theory. Englewood Cliffs, NJ: Prentice Hall.

[ref4] Beyens I. Valkenburg P. M. (2019). Parental media mediation in adolescence: a comparative study of parent and adolescent reports. J. Broadcast. Electron. Media 63, 716–736. doi: 10.1080/08838151.2019.1680071

[ref5] Bhargava V. R. Velasquez M. (2021). Ethics of the attention economy: the problem of social media addiction. Bus. Ethics Q. 31, 321–359. doi: 10.1017/beq.2020.32

[ref6] Boniel-Nissim M. Efrati Y. Dolev-Cohen M. (2020). Parental mediation regarding children’s pornography exposure: the role of parenting style, protection motivation and gender. J. Sex Res. 57, 42–51. doi: 10.1080/00224499.2019.1590795, PMID: 30925073

[ref7] Brehm S. S. Brehm J. W. (2013). Psychological reactance: a theory of freedom and control” is San Diego, California, USA.

[ref8] Busch P. A. McCarthy S. (2021). Antecedents and consequences of problematic smartphone use: a systematic literature review of an emerging research area. Comput. Hum. Behav. 114:106414. doi: 10.1016/j.chb.2020.106414

[ref9] Charlot Colomès A. A. Duchesne S. Châteauvert B. (2021). Autonomy support and school adjustment: the mediating role of basic psychological needs. Int. J. Sch. Educ. Psychol., 9: S182–S200. doi: 10.1080/21683603.2021.1877226

[ref10] Chen Y. (2021). The mechanism of the effect of parental mediation on adolescent mobile phone addiction intervention study. PhD thesis.: Central China Normal University.

[ref11] Chen X. Lin Y. Liu Q. X. (2020). Technoference and adolescent smartphone addiction: the effect of core self-evaluations and need satisfaction perceived online. J. Psychol. Sci. 43, 355–362. doi: 10.16719/j.cnki.1671-6981.20200214

[ref12] Çiçek I. Tanrıverdi S. Şanlı M. E. Bulus M. (2021). Parental attitudes and socio-demographic factors as predictors of smartphone addiction in university students. Int. J. Psychol. Educ. Stud. 8, 158–169. doi: 10.52380/ijpes.2021.8.2.430

[ref13] Ciydem E. Avci D. Uyar M. Seyhan A. (2023). The relationship between basic psychological needs and emotional and behavioral problems in middle school students. J. Child Adolesc. Psychiatr. Nurs. 36, 179–187. doi: 10.1111/jcap.12411, PMID: 36727582

[ref14] Clark L. S. (2011). Parental mediation theory for the digital age. Commun. Theory 21, 323–343. doi: 10.1111/j.1468-2885.2011.01391.x

[ref15] Da Silva Pinho A. Céspedes Izquierdo V. Lindström B. van den Bos W. (2024). Youths' sensitivity to social media feedback: a computational account. Sci. Adv. 10:eadp8775. doi: 10.1126/sciadv.adp8775, PMID: 39441931 PMC11498218

[ref16] Davis R. A. (2001). A cognitive-behavioral model of pathological internet use. Comput. Human Behav. 17, 187–195. doi: 10.1016/S0747-5632(00)00041-8

[ref17] Deci E. L. Ryan R. M. (2000). The “what” and “why” of goal pursuits: human needs and the self-determination of behavior. Psychol. Inq. 11, 227–268. doi: 10.1207/S15327965PLI1104_01

[ref18] Dedkova L. Smahel D. (2019). Online parental mediation: associations of family members’ characteristics to individual engagement in active mediation and monitoring. J. Fam. Issues 41, 1112–1136. doi: 10.1177/0192513X19888255

[ref19] Durak A. Kaygin H. (2020). Parental mediation of young children’s internet use: adaptation of parental mediation scale and review of parental mediation based on the demographic variables and digital data security awareness. Educ. Inf. Technol. 25, 2275–2296. doi: 10.1007/s10639-019-10079-1

[ref20] Fabio R. A. Stracuzzi A. Faro R. (2022). Problematic smartphone use leads to behavioral and cognitive self-control deficits. Int. J. Environ. Res. Public Health 19:7445. doi: 10.3390/ijerph19127445 35742695 PMC9223448

[ref21] Fu X. Liu J. Liu R. Ding Y. Hong W. Jiang S. (2020). The impact of parental active mediation on adolescent mobile phone dependency: a moderated mediation model. Comput. Human Behav. 107:106280. doi: 10.1016/j.chb.2020.106280

[ref22] Gao Q. Zheng H. Sun R. Lu S. (2022). Parent-adolescent relationships, peer relationships, and adolescent mobile phone addiction: the mediating role of psychological needs satisfaction. Addict. Behav. 129:107260. doi: 10.1016/j.addbeh.2022.107260, PMID: 35151093

[ref23] Gong X. Wang C. (2023). Interactive effects of parental psychological control and autonomy support on emerging adults’ emotion regulation and self-esteem. Curr. Psychol. 42, 16111–16120. doi: 10.1007/s12144-021-01483-3

[ref24] Gu H. Chen W. Cheng Y. (2024). Longitudinal relationship between harsh parenting and adolescent non-suicidal self-injury: the roles of basic psychological needs frustration and self-concept clarity. Child Abuse Negl. 149:106697. doi: 10.1016/j.chiabu.2024.106697, PMID: 38412590

[ref25] Hong F. Chiu S. Huang D. (2012). A model of the relationship between psychological characteristics, mobile phone addiction and use of mobile phones by Taiwanese university female students. Comput. Human Behav. 28, 2152–2159. doi: 10.1016/j.chb.2012.06.020

[ref26] Jin Y. Jiang S. (2025). Theoretical perspectives on adolescent internet addiction: a comprehensive literature review. Health Soc. Care Community 2025:4875332. doi: 10.1155/hsc/4875332

[ref27] Kapetanovic S. Nielsen M. D. André F. Gurdal S. Claesdotter-Knutsson E. (2025). Exploring parent-child relationships in a Swedish child and adolescent psychiatry – cohort of adolescents with internet gaming disorder. BMC Psychol. 13:18. doi: 10.1186/s40359-024-02306-3, PMID: 39780294 PMC11708115

[ref28] Kardefelt-Winther D. (2014). A conceptual and methodological critique of internet addiction research: towards a model of compensatory internet use. Comput. Hum. Behav. 31, 351–354. doi: 10.1016/j.chb.2013.10.059

[ref29] Ladani H. M. Yogesh M. Trivedi N. S. Gandhi R. B. Lakkad D. (2025). Exploring smartphone utilization patterns, addiction, and associated factors in school-going adolescents: a mixed-method study. J. Fam. Med. Prim. Care 14, 334–340. doi: 10.4103/jfmpc.jfmpc_1308_24, PMID: 39989525 PMC11845006

[ref30] Li Q. Liu Z. (2025). Parental psychological control and adolescent smartphone addiction: roles of reactance and resilience. BMC Psychol. 13:139. doi: 10.1186/s40359-025-02477-7, PMID: 39972515 PMC11841267

[ref9001] Li D. Zhang W. Li X. Zhen S. Wang Y. (2010). Stressful life events and problematic Internet use by adolescent females and males: A mediated moderation model. Comput. Hum. Behav. 26:1199–1207. doi: 10.1016/j.chb.2010.03.031, PMID: 37622819

[ref31] Li D. P. Zhou Y. Y. Zhao L. Y. Wang Y. H. Sun W. Q. (2016). Cumulative ecological risk and adolescent internet addiction: the mediating role of basic psychological need satisfaction and positive outcome expectancy. Acta Psychol. Sin. 48, 1519–1537. doi: 10.3724/SP.J.1041.2016.01519

[ref33] Liu Y. Huang X. (2021). Effects of basic psychological needs on resilience: a human agency model. Front. Psychol. 12:700035. doi: 10.3389/fpsyg.2021.700035, PMID: 34531790 PMC8438124

[ref34] Liu Z. Li M. Ren C. Zhu G. Zhao X. (2022). Relationship between physical activity, parental psychological control, basic psychological needs, anxiety, and mental health in Chinese engineering college students during the COVID-19 pandemic. Front. Psychol. 13:802477. doi: 10.3389/fpsyg.2022.802477, PMID: 35350737 PMC8958037

[ref35] Liu J. Wu L. Sun X. Bai X. Duan C. (2023). Active parental mediation and adolescent problematic internet use: the mediating role of parent–child relationships and hiding online behavior. Behav. Sci. 13:679. doi: 10.3390/bs13080679, PMID: 37622819 PMC10451844

[ref36] Martela F. Lehmus-Sun A. Parker P. D. Pessi A. B. Ryan R. M. (2022). Needs and well-being across Europe: basic psychological needs are closely connected with well-being, meaning, and symptoms of depression in 27 European countries. Soc. Psychol. Personal. Sci. 14, 501–514. doi: 10.1177/19485506221113678

[ref37] Martín-Cárdaba M. Á. Martínez Díaz M. V. Lafuente Pérez P. García Castro J. (2024). Smartphone ownership, minors’ well-being, and parental mediation strategies. An analysis in the context of social media influencers. J. Youth Adolesc. 53, 2202–2218. doi: 10.1007/s10964-024-02013-7, PMID: 38782845 PMC11413121

[ref38] Mayerhofer D. Haider K. Amon M. Gächter A. O'Rourke T. Dale R.. (2024). The association between problematic smartphone use and mental health in Austrian adolescents and young adults. Healthcare 12:600. doi: 10.3390/healthcare12060600 38540564 PMC10970667

[ref39] Nathanson A. I. (2002). The unintended effects of parental mediation of television on adolescents. Media Psychol. 4, 207–230. doi: 10.1207/S1532785XMEP0403_01

[ref40] Nikken P. Jansz J. (2014). Developing scales to measure parental mediation of young children's internet use. Learn. Media Technol. 39, 250–266. doi: 10.1080/17439884.2013.782038

[ref41] Panova T. Carbonell X. (2018). Is smartphone addiction really an addiction? J. Behav. Addict. 7, 252–259. doi: 10.1556/2006.7.2018.49, PMID: 29895183 PMC6174603

[ref42] Paterna A. Alcaraz-Ibáñez M. Aguilar-Parra J. M. Salavera C. Demetrovics Z. Griffiths M. D. (2024). Problematic smartphone use and academic achievement: a systematic review and meta-analysis. J. Behav. Addict. 13, 313–326. doi: 10.1556/2006.2024.00014, PMID: 38669081 PMC11220804

[ref43] Peng S. Niu G. F. Wang X. Zhang H. P. Hu X. E. (2021). Parental autonomy support and adolescents' positive emotional adjustment: mediating and moderating roles of basic need satisfaction. Psychol. Dev. Educ. 37, 240–248. doi: 10.16187/j.cnki.issn1001-4918.2021.02.11

[ref44] Podsakoff P. M. Mac Kenzie S. B. Lee J. Podsakoff N. P. (2003). Common method biases in behavioral research: a critical review of the literature and recommended remedies. J. Appl. Psychol. 88, 879–903. doi: 10.1037/0021-9010.88.5.879 14516251

[ref45] Ru Y. Norlizah H. C. Nasuha Burhanuddin N. A. Liu H. Dong J. (2025). The correlation between mindfulness and problematic smartphone use: a meta-analysis. Addict. Behav. 164:108272. doi: 10.1016/j.addbeh.2025.108272, PMID: 39923383

[ref46] Sun R. Gao Q. Xiang Y. (2022). Perceived parental monitoring of smartphones and problematic smartphone use in adolescents: mediating roles of self-efficacy and self-control. Cyberpsychol. Behav. Soc. Netw. 25, 784–792. doi: 10.1089/cyber.2022.0040, PMID: 36409521

[ref47] Sun H. Tang K. (2025). Psychometric evaluation and measurement invariance of the problematic smartphone use scale among college students: a national survey of 130, 145 participants. Addiction 120, 629–641. doi: 10.1111/add.16699, PMID: 39505322

[ref48] Tie B. Zhang T. He M. Geng L. Feng Q. Liu C.. (2025). Smartphone and the brain: stress and self-control mediate the association between the connectome-based predictive modeling of fMRI brain network and problematic smartphone use. Comput. Human Behav. 165:108531. doi: 10.1016/j.chb.2024.108531

[ref49] Vossen H. G. M. van den Eijnden R. J. J. M. Visser I. Koning I. M. (2024). Parenting and problematic social media use: a systematic review. Curr. Addict. Rep. 11, 511–527. doi: 10.1007/s40429-024-00559-x

[ref50] Wacks Y. Weinstein A. M. (2021). Excessive smartphone use is associated with health problems in adolescents and Young adults. Front. Psych. 12:669042. doi: 10.3389/fpsyt.2021.669042, PMID: 34140904 PMC8204720

[ref52] West M. Rice S. Vella-Brodrick D. (2023). Mid-adolescents’ social media use: supporting and suppressing autonomy. J. Adolesc. Res. 40, 448–482. doi: 10.1177/07435584231168402

[ref53] Yang J. Fu X. Liao X. Li Y. (2020). Association of problematic smartphone use with poor sleep quality, depression, and anxiety: a systematic review and meta-analysis. Psychiatry Res. 284:112686. doi: 10.1016/j.psychres.2019.112686, PMID: 31757638

[ref54] Yin B. Shen Y. (2023). Compensatory beliefs in the internet gratification behavior: a study of game-based assessment. Front. Public Health 11:997108. doi: 10.3389/fpubh.2023.997108, PMID: 36761132 PMC9902763

[ref55] Young K. S. (1998). Internet addiction: the emergence of a new clinical disorder. Cyber Psychol. Behav. 1, 237–244. doi: 10.1089/cpb.1998.1.237

[ref56] Yu C. F. Zhang W. Zeng Y. Y. Ye T. Hu J. P. Li D. L. (2012). Gratitude, basic psychological needs, and problematic internet use in adolescence. Psychol. Dev. Educ. 28, 83–90. doi: 10.16187/j.cnki.issn1001-4918.2012.01.005

